# Effect of Patient's Personality on Satisfaction with Their Present Complete Denture and after Increasing the Occlusal Vertical Dimension: A Study of Edentulous Egyptian Patients

**DOI:** 10.1155/2014/635943

**Published:** 2014-07-08

**Authors:** Shaimaa M. Fouda, Mohamed S. Al-Attar, Jorma I. Virtanen, Aune Raustia

**Affiliations:** ^1^Department of Substitutive Dental Sciences, College of Dentistry, Dammam University, P.O. Box 1982, Dammam 31411, Saudi Arabia; ^2^Department of Prosthodontics, Faculty of Dentistry, Alexandria University, P.O. Box 21500, Alexandria, Egypt; ^3^Department of Community Dentistry, Institute of Dentistry, University of Oulu, P.O. Box 5281, 90014 Oulu, Finland; ^4^Oral and Maxillofacial Department, Oulu University Hospital, Finland; ^5^Department of Prosthetic Dentistry and Stomatognathic Physiology, Institute of Dentistry, University of Oulu, P.O. Box 5281, 90014 Oulu, Finland

## Abstract

Complete denture wearers often find it difficult to accept a new denture. Personality traits are among the factors that possibly affect patient satisfaction with a complete denture. Our aim was to investigate the influence of patients' personality on satisfaction with their present denture and after an increase in the occlusal vertical dimension (OVD). Sixty edentulous patients with complete dentures (22 men and 38 women, mean age 66 years, and range 50–75 years) participated in the study. The age of their complete dentures ranged from 5 to 16 years. Patients' personalities were evaluated using the Arabic version of the Eysenck Personality Questionnaire. Their satisfaction with their dentures before and after restoration of the OVD and relining of the mandibular denture was evaluated using two questionnaires (I and II), Patients with a high score of neuroticism were less satisfied with their original dentures and after relining and an increase of OVD compared with patients with an average score in that trait. The personality trait of psychoticism was significant to patients' acceptance of an increase in OVD; that is, patients with a high score were less satisfied with their dentures after increase of OVD than patients with an average score. It is concluded that personality traits affect patients' acceptance of their complete dentures.

## 1. Introduction

The prevalence of edentulism has decreased during the last decades, especially in developed countries. However, the level of edentulism is still high in developing countries [1–3]. In addition, more people worldwide are advancing into old age and a growing number of edentulous people are expected [1]. Edentulism has a deep impact on the quality of life, affecting individuals' physiological, biological, social, and psychological state [4]. It can also cause a state of depression due to disturbances in speech, esthetics, mastication, and a feeling of inferiority because an important part of the person has been lost [5].

Conventional complete dentures are still the treatment of choice in many cases for both economic and biological reasons [6]. However, a considerable proportion of denture wearers are dissatisfied with their complete dentures [7]. Several studies investigated factors that may affect patients' satisfaction with their complete dentures, such as denture technical quality, condition of the residual ridges, and patient's gender, age, previous denture experience, and personality [5, 6, 8]. Conflicting results have been reported regarding associations with denture acceptance; yet emotional and psychological factors seem to play an important role in acceptance of complete dentures [4, 5]. The use of a removable denture depends on the patient's ability to adapt to the function of the dentures as well as adaptation at the emotional level [5, 6].

The relationship between patient's personality and denture satisfaction has been investigated and inconsistent findings were reported, regarding the relationship between personality and denture satisfaction [4–6, 9]. Several questionnaires have been used to assess patients' personality, for example, the shortened version of the Minnesota Multiphasic Personality Inventory (MMPI) [9], the Cornell Medical Index (CMI) [10], Cattell's 16PFQ form C questionnaire [11], the Revised Personality Inventory (NEO PI-R) [5], the Eysenck Short Scale Personality Questionnaire (EPQ-R) [12–15], and the Eysenck Personality Inventory (EPI) [16]. It has been noted that some personality traits like neuroticism influence patient satisfaction with a removable denture [5, 10–12, 16, 17].

Long-time complete denture wear often results in reduced occlusal vertical dimension due to residual ridge resorption and acrylic tooth wear. This is especially true for the mandible [18]. Restoring the OVD is an important treatment procedure in prosthetic treatment, especially in edentulous patients [19].

The aim of our study was to investigate the effect of the patients' personality on satisfaction with their present complete dentures and after an increase in the OVD by using the Arabic version of the EPQ for personality assessment. The hypothesis of the study was that personality traits were expected to have an effect on the patients' satisfaction with the present dentures and after an increase in the OVD.

## 2. Materials and Methods

Sixty completely edentulous patients with maxillary and mandibular complete dentures in use (22 men and 38 women, mean age 66 years, and range 50–75 years) who were seeking prosthetic treatment for relining of their old dentures or fabrication of new ones were selected from the Department of Prosthodontics, Faculty of Dentistry, Alexandria University, Egypt, in 2009–2011. All patients who had complete dentures with a reduced OVD and an interocclusal rest space of no more than 10 mm participated in the study after giving their informed consent. The age of their complete dentures ranged from 5 to 16 years (mean 9 years). The patients included had one or more mild signs of temporomandibular disorders (TMD), that is, masticatory muscle pain during palpation or headache. Patients with temporomandibular joint (TMJ) clicking and patients with a significant restriction in opening movement of the mandible were excluded [20]. Patients with a systemic disease that possibly affects the masticatory system, for example, rheumatoid arthritis or epilepsy, were also excluded from the study. The study was approved by the Research Ethical Committee, Faculty of Dentistry, Alexandria University.

**Figure 1:**
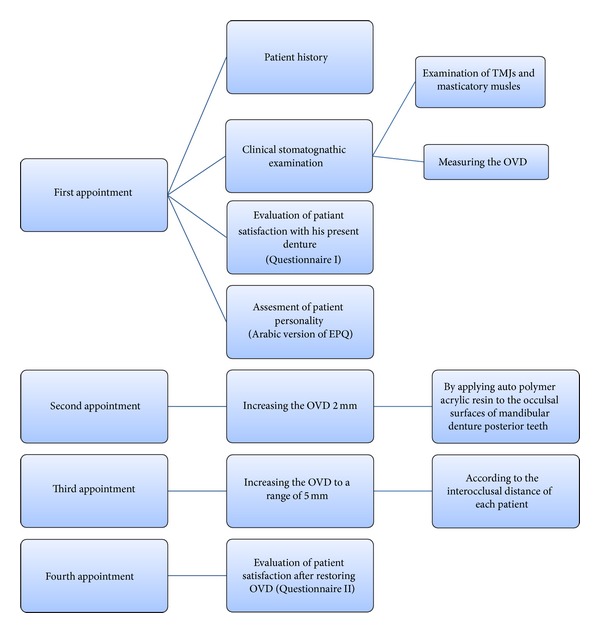
Patients were recalled for three visits after first appointment at two-week intervals (OVD: occlusal vertical dimension).

The patients were recalled for three visits after the first visit at two-week intervals (see Figure 1). All sixty patients completed the study.

### 2.1. The First Visit

Patient history was recorded. A clinical stomatognathic examination was performed, including measurement of the opening movement of the mandible and recording of TMJ sounds, TMJ pain, and/or pain in the masticatory muscles during palpation [21]. The interocclusal rest space was determined by calculating the difference between rest vertical dimension determined phonetically and OVD with the two-dot technique using a ruler [22]. The patients' personality was assessed by using the Arabic version of the Eysenck Scale Personality Questionnaire [23]. All the patients were interviewed by the same psychologist, who presented the questionnaire to the patients and recorded their answers. The questionnaire consisted of 90 questions with yes/no answers, and it yielded scores for each of the major personality traits: psychoticism, extrovertism, neuroticism, criminality, and lie scale. The patient's attitude toward his/her present dentures before relining and restoration of the OVD was determined by asking questions related to chewing efficiency, speech, and overall satisfaction (Questionnaire I) [24]. Chair-side relining of poorly fitting mandibular denture was done using self-curing acrylic resin (Hardliner, PROMEDICA, Neumünster, Germany) [22, 25].

### 2.2. The Second and Third Visits

The OVD was restored gradually, 2 mm during the second visit and up to 5 mm during the third visit, according to the interocclusal rest space of each patient, by applying autopolymer acrylic resin to the occlusal surfaces of the posterior teeth of the mandibular denture [18] (SNAP Parkell, NY, USA).

### 2.3. The Fourth Visit

Evaluation of the patients' denture satisfaction after restoration of the OVD was assessed using a second comparative questionnaire (Questionnaire II) [24]. An independent interviewer, who did not participate in the clinical examination or the treatment procedures, presented Questionnaires I and II to the patients. The interviewer read each question to the patient and recorded the patient's answers. All the clinical examinations and treatment procedures were performed by the same dentist specialized in prosthetic dentistry (SMF).

### 2.4. Statistical Analysis

The Statistical Package for Social Sciences (SPSS/version 17) software was used for data analysis. The Chi-square test served for analysis of categorical data and comparison of the patients' satisfaction and different personality traits.

## 3. Results

Table 1 shows the patients' overall satisfaction with their complete dentures at the first appointment. Two-thirds (73%) of the patients showed slight to complete dissatisfaction with their dentures. Most of the patients (83%) used their dentures always/almost always for eating, although 70% of them mostly/frequently experienced chewing discomfort and 67% rarely felt it easy to chew hard food. Over half (53%) of the patients experienced pain in their jaws and 58% reported jaw aching or stiffness in the morning. After restoration of the OVD and denture relining, the patients' perception of their dentures showed improvement in chewing ability (67%) and chewing comfort (53%), and they perceived also improvement in chewing hard food (52%) (Table 2). The patients also reported improvement in jaw aching or stiffness in the morning (27 patients) as well as in jaw pain (19 patients).

When the levels of satisfaction with the original dentures before and after dentures relining and restoration of the OVD were compared, a significant increase in the patients' satisfaction was observed (*P* < 0.05) (Table 3). Neuroticism correlated with the patients' satisfaction with their original dentures before and after restoration of the OVD and relining (*P* < 0.05). Two-thirds (29/44, 66%) of the patients who were unsatisfied with their original denture had a high score on neuroticism, while all the satisfied patients had an average neuroticism score (Table 4). No correlation between personality traits of psychoticism and extrovertism and the patients' satisfaction with their original denture was seen (Table 4). After an increase in the OVD and relining, a significant association between the level of the patients' satisfaction and psychoticism was observed (*P* < 0.05). Of the unsatisfied patients (*n* = 12), 10 had a high score on psychoticism (Table 5). After denture relining and restoration of the OVD, 12 patients were unsatisfied with their dentures and nine of them had a high score on neuroticism (Table 5).

## 4. Discussion

In the present study a significant increase in the patients' satisfaction after chair-side relining of poorly fitting mandibular dentures and restoration of the OVD was observed. Our results confirm the findings of previous studies showing that dissatisfaction with dentures in addition to the retention and stability of the dentures is maybe associated with emotional instability or neuroticism [10–12, 16, 17]. Psychological factors can affect patients' acceptance of a denture, and therefore a personality assessment could be beneficial in predicting satisfaction with a complete denture [5].

This result agrees with the finding of the previous studies, which found that most patients with a modified denture perceived improvement or no change [24, 26]. Most patients with old dentures accepted a gradual increase in the OVD, even if they were used to their old dentures with a reduced OVD. This could also be explained by the improvement in chewing functions and the gradual increase in the OVD performed in this study, which might be tolerated better by the patients than an increase in one step [22].

The personality trait of psychoticism was not significantly related to patients' satisfaction with their original dentures. This was also reported in earlier studies [12, 27]. However, a high psychoticism score was related to patients' dissatisfaction after relining and restoration of the OVD of their old dentures. Psychoticism measures tough-mindedness. A person with a high psychoticism score tends to behave impulsively, being insensitive, aggressive, and finding it difficult to adapt [28, 29]. Therefore, psychoticism had no effect on patients' satisfaction with their old dentures because they were used to them, while it affected their acceptance of a change in their dentures, that is, an increase in the OVD. Another explanation might be the evaluation of the patients' satisfaction with the increased OVD after only two weeks, which is a short period for patients with a high psychoticism score to adapt to a change in their dentures. This finding is in line with a study that found a presence of a difference in the relationship between personality profiles and satisfaction before and after treatment [30].

We found a significant relationship between high neuroticism scores and patient dissatisfaction. Patient neuroticism has been suggested as a negative indicator of complete denture success [31]. A person with a high neuroticism score has been described as being a worrying individual, frequently depressed, and overtly emotional and having a greater tendency to complain about his/her denture [27, 28]. Some studies have shown a significant negative association between neuroticism and patient satisfaction with a denture [5, 10, 12, 16]. In addition, vague denture complaints and esthetic complaints about a bulbous face have been found to be related to neuroticism [23]. It has been noted that neuroticism remained the only trait that maintained a relationship with patients' satisfaction before and after treatment, while other personality profiles were found to be significantly related to satisfaction ratings after treatment [30]. Other studies found that dissatisfied patients may possess particular personality traits that influence their satisfaction with dentures. Although in these studies the personality trait of neuroticism was not measured, the findings are still in line with our results [4, 11].

Extroversion was not found to have a significant effect on the level of satisfaction. An extravert is characterized by being sociable, talkative, outgoing, cheerful, and optimistic; in contrast an introvert is overly careful, shy, pessimistic, and calm and dislikes company [28]. This result may be related to the relatively small number of patients with a low extroversion score. However, this finding agrees with the results of previous studies which suggested that extrovertism was not related to patient satisfaction with dentures [12, 27]. This finding disagrees with a study that found a relationship between the personality trait of extrovertism and functional complaints about the mandibular denture and complaints about a hollow face [17].

The Arabic version of the EPQ for assessment of patients' personality was utilized because of its validity and reliability in Egypt [23]. The EPQ is an effective personality measurement tool which analyzes the characteristics of personality structure [28]. The strengths of our study were that the personality assessments were done by the same psychologist, the questionnaires were presented to all the patients by the same interviewer, all the clinical procedures were performed by the same dentist, and all the patients completed the study. The limitations include a relatively small sample size and a lack of patients with a high extrovertism score, since the patients here do not represent the whole population.

## 5. Conclusion

It is concluded that personality traits affect patients' acceptance of a complete denture. Patients with a high score on neuroticism were less satisfied with their original dentures and after relining and an increase in the OVD compared with patients with an average score on that trait. A high psychoticism score was found to be related to patients' dissatisfaction after restoration of the OVD.

## Figures and Tables

**Table 1:** Patients' (*n* = 60) satisfaction with their original complete dentures based on their answers to Questionnaire I.

Question	Mostly	Frequently	Occasionally	Rarely
(1) Eating with dentures	83%	17%	0%	0%
(2) Experience chewing discomfort	62%	8%	30%	0%
(3) Ease of chewing hard food	0%	10%	23%	67%
(4) Eating enjoyment	48%	20%	17%	15%
(5) Affect food choices	68%	8%	14%	10%
(6) Food particles under dentures	91%	7%	2%	0%
(7) Taste (difference)	33%	38%	17%	12%
(8) Speech (difficulties)	36%	25%	22%	17%
(9) Dentures odor changed	37%	20%	17%	26%
(10) Cleaning difficulties	25%	17%	25%	33%
(11) Cleanliness	20%	22%	25%	33%
(12) Security	8%	12%	17%	63%
(13) Satisfaction				
Slightly, moderately, or completely satisfied	27%			
Slightly, moderately, or completely dissatisfied	73%			
(14) Jaw ache or stiffness mourning	13%	20%	25%	42%
(15) Pain in jaw	20%	13%	20%	47%

**Table 2:** Patients' (*n* = 60) satisfaction with their complete dentures after restoring OVD and relining based on their answers to Questionnaire II.

Question	−3	−2	−1	0	1	2	3
(1) Chewing ability	13.3%	3.3%	13.3%	3.3%	20.0%	16.7%	30.0%
(2) Chewing comfort	16.7%	5.0%	20.0%	5.0%	25.0%	18.3%	10.0%
(3) Ease of chewing hard food	18.3%	3.3%	16.7%	10.0%	20.0%	16.7%	15.0%
(4) Food choices	11.7%	3.3%	16.7%	8.3%	16.7%	25.0%	18.3%
(5) Eating enjoyment	11.7%	6.7%	11.7%	5.0%	20.0%	21.7%	23.3%
(6) Cleaning	5.0%	10.0%	28.3%	8.3%	13.3%	15.0%	20.0%
(7) Cleanliness	10.0%	5.0%	18.3%	16.7%	15.0%	13.3%	21.7%
(8) Odor	8.3%	5.0%	16.7%	26.7%	16.7%	16.7%	10.0%
(9) Odor frequency	13.3%	6.7%	16.7%	15.0%	16.7%	8.3%	23.3%
(10) Security	6.7%	3.3%	20.0%	6.7%	13.3%	13.3%	36.7%
(11) Speech	10.0%	8.3%	18.3%	21.7%	10.0%	11.7%	20.0%
(12) Jaw ache or stiffness	11.7%	5.0%	21.7%	16.7%	5.0%	5.0%	35.0%
(13) Pain in jaws	16.7%	10.0%	16.7%	25.0%	8.3%	8.3%	15.0%

**Table 3:** Patients' (*n* = 60) satisfaction with their original complete dentures, and after relining and restoration of the OVD.

Level of satisfaction	Original denture		After restoring VDO		*P* value
	Number	%	Number	%	
Satisfied	6	10	33	55	0.0013
Neutral	10	17	15	25	
Unsatisfied	44	73	12	20	

**Table 4:** Association between the level of patients' satisfaction with their present complete dentures and psychoticism, neuroticism, and extroversion.

	Satisfied		Neutral		Unsatisfied		Total (*n*)	*P* value
	*N*	%	*N*	%	*N*	%		
Psychoticism								
Low score	0	0	0	0.0	0	0.0	0	0.21
Average score	6	20	2	7	22	73	30	
High score	0	0	8	27	22	73	30	
Total (*n*)	**6**		** 10**		**44**			
Neuroticism								
Low score	0	0	0	0	0	0	0	0.005
Average score	6	24	4	16	15	60	25	
Highs core	0	0	6	17	29	83	35	
Total (*n*)	** 6**		** 10**		** 44**			
Extroversion								
Low score	0	0	2	17	10	83	12	0.233
Average score	6	13	8	17	34	71	48	
High score	0	0	0	0	0	0	0	
Total (*n*)	** 6**		** 10**		** 44**			

Average scores on

psychoticism: males (M) = 3.95 (±3.28) and females (F) = 2.77 (±2.54);

neuroticism: M = 9.67 (±5.10) and F = 12.73 (±5.07);

extroversion: M = 13.12 (±4.95) and F = 12.95 (±4.67).

**Table 5:** Association between the level of patients' satisfaction after relining and restoration of the OVD of their complete dentures and psychoticism, neuroticism, and extroversion.

	Satisfied		Neutral		Unsatisfied		Total (*n*)	*P* value
	*N*	%	*N*	%	*N*	%		
Psychoticism								
Low score	0	0	0	0	0	0.0	0	0.032
Average score	19	63	9	30	2	7	30	
High score	14	47	6	20	10	33	30	
Total (*n*)	** 33**		** 15**		** 12**			
Neuroticism								
Low score	0	0	0	0	0	0	0	0.039
Average score	20	80	2	8	3	12	25	
High score	13	37	13	37	9	26	35	
Total (*n*)	** 33**		** 15**		** 12**			
Extroversion								
Low score	10	83	0	0	2	17	12	0.225
Average score	23	48	15	31	10	21	48	
High score	0	0	0	0	0	0	0	
Total (*n*)	**33**		** 15**		** 12**			
